# The Type I Interferon Response Determines Differences in Choroid Plexus Susceptibility between Newborns and Adults in Herpes Simplex Virus Encephalitis

**DOI:** 10.1128/mBio.00437-16

**Published:** 2016-04-12

**Authors:** Douglas R. Wilcox, Stephen S. Folmsbee, William J. Muller, Richard Longnecker

**Affiliations:** a Department of Microbiology-Immunology, Northwestern University Feinberg School of Medicine, Chicago, Illinois, USA; b Department of Medicine, Northwestern University Feinberg School of Medicine, Chicago, Illinois, USA; c Department of Pediatrics, Northwestern University Feinberg School of Medicine, Chicago, Illinois, USA

## Abstract

Newborns are significantly more susceptible to severe viral encephalitis than adults, with differences in the host response to infection implicated as a major factor. However, the specific host signaling pathways responsible for differences in susceptibility and neurologic morbidity have remained unknown. In a murine model of HSV encephalitis, we demonstrated that the choroid plexus (CP) is susceptible to herpes simplex virus 1 (HSV-1) early in infection of the newborn but not the adult brain. We confirmed susceptibility of the CP to HSV infection in a human case of newborn HSV encephalitis. We investigated components of the type I interferon (IFN) response in the murine brain that might account for differences in cell susceptibility and found that newborns have a dampened interferon response and significantly lower basal levels of the alpha/beta interferon (IFN-α/β) receptor (IFNAR) than do adults. To test the contribution of IFNAR to restricting infection from the CP, we infected IFNAR knockout (KO) adult mice, which showed restored CP susceptibility to HSV-1 infection in the adult. Furthermore, reduced IFNAR levels did not account for differences we found in the basal levels of several other innate signaling proteins in the wild-type newborn and the adult, including protein kinase R (PKR), that suggested specific regulation of innate immunity in the developing brain. Viral targeting of the CP, a region of the brain that plays a critical role in neurodevelopment, provides a link between newborn susceptibility to HSV and long-term neurologic morbidity among survivors of newborn HSV encephalitis.

## IMPORTANCE

Compared to adults, newborns are significantly more susceptible to severe disease following HSV infection. Over half of newborn HSV infections result in disseminated disease or encephalitis, with long-term neurologic morbidity in 2/3 of encephalitis survivors. We investigated differences in host cell susceptibility between newborns and adults that contribute to severe central nervous system disease in the newborn. We found that, unlike the adult brain, the newborn choroid plexus (CP) was susceptible early in HSV-1 infection. We demonstrated that IFN-α/β receptor levels are lower in the newborn brain than in the adult brain and that deletion of this receptor restores susceptibility of the CP in the adult brain. The CP serves as a barrier between the blood and the cerebrospinal fluid and plays a role in proper neurodevelopment. Susceptibility of the newborn choroid plexus to HSV-1 has important implications in viral spread to the brain and, also, in the neurologic morbidity following HSV encephalitis.

## INTRODUCTION

Newborns are particularly susceptible to a wide range of viral encephalitides compared to adults. Herpes simplex virus (HSV) is the most common cause of viral encephalitis, but infection with HSV in the adult typically results in asymptomatic acquisition or benign mucosal disease, and only rarely results in encephalitis (1). This is in stark contrast to HSV infection in the newborn, where more than 30% of those infected progress to encephalitis (2). Among survivors, 2/3 will go on to have permanent neurologic morbidity (2). The disparate outcomes between adults and newborns following HSV infection suggest an age-dependent difference in susceptibility to disease based on host factors.

The unique immune responses in the newborn are partly reflections of the dramatic shift from a sterile uterine environment, microbial colonization of organs, and the role of cytokine and chemokine balance for proper neurodevelopment. In addition to the well-described differences in the newborn adaptive immune response (3), evidence suggests that there are also important differences in the innate response between age groups (4). Although the type I interferon (IFN) response reduces viral replication and improves survival in the adult (5), it does not contribute to survival following HSV infection in the newborn brain (4). However, the type I IFN response is not completely absent in the newborn brain, since it does contribute to survival following infection with a recombinant HSV-1 deficient for interaction with proteins in the host response pathways (6). Whether this observation results from a global down-regulation of IFN signaling components in the brain or specific key regulators in the pathway remains unknown.

HSV belongs to the family of neurotropic alphaherpesviruses, and their infection of neurons in the adult brain has been well described (7). However, some virus families which are not neurotropic in the adult brain target neural progenitor cells and glial cells in the newborn brain (8). This suggests possible age-dependent changes in neurotropism. The choroid plexus (CP), found throughout the ventricles and responsible for cerebrospinal fluid (CSF) production in the brain, has recently been described as a target for several different pathogens (9). Distinct from the blood-brain barrier (BBB), which is comprised of endothelial cells in association with pericytes, astrocytes, and the basement membrane and forms a contiguous membrane barrier, the specialized CP epithelium surrounds fenestrated capillaries to form the blood-CSF barrier. Importantly, the CP is a site of lymphocytic infiltration into the brain via the blood-CSF barrier (10) and may also provide access to the brain for pathogens. Beyond its role in CSF production, the CP secretes several factors that influence neural progenitors and brain development in the subventricular zone (SVZ) (11). Insult to the CP can have a profound impact on neurodevelopment, and prior studies have shown that peripheral inflammation can dramatically change the transcriptome and secretome of the CP, altering fetal ventricular zone proliferation and, ultimately, cortical layer formation (12, 13).

In the current study, we hypothesized that host cell susceptibility during HSV encephalitis is different in the newborn than in the adult and that age-dependent differences in the innate response to infection contribute to a change in neurotropism.

## RESULTS AND DISCUSSION

**FIG 1:**
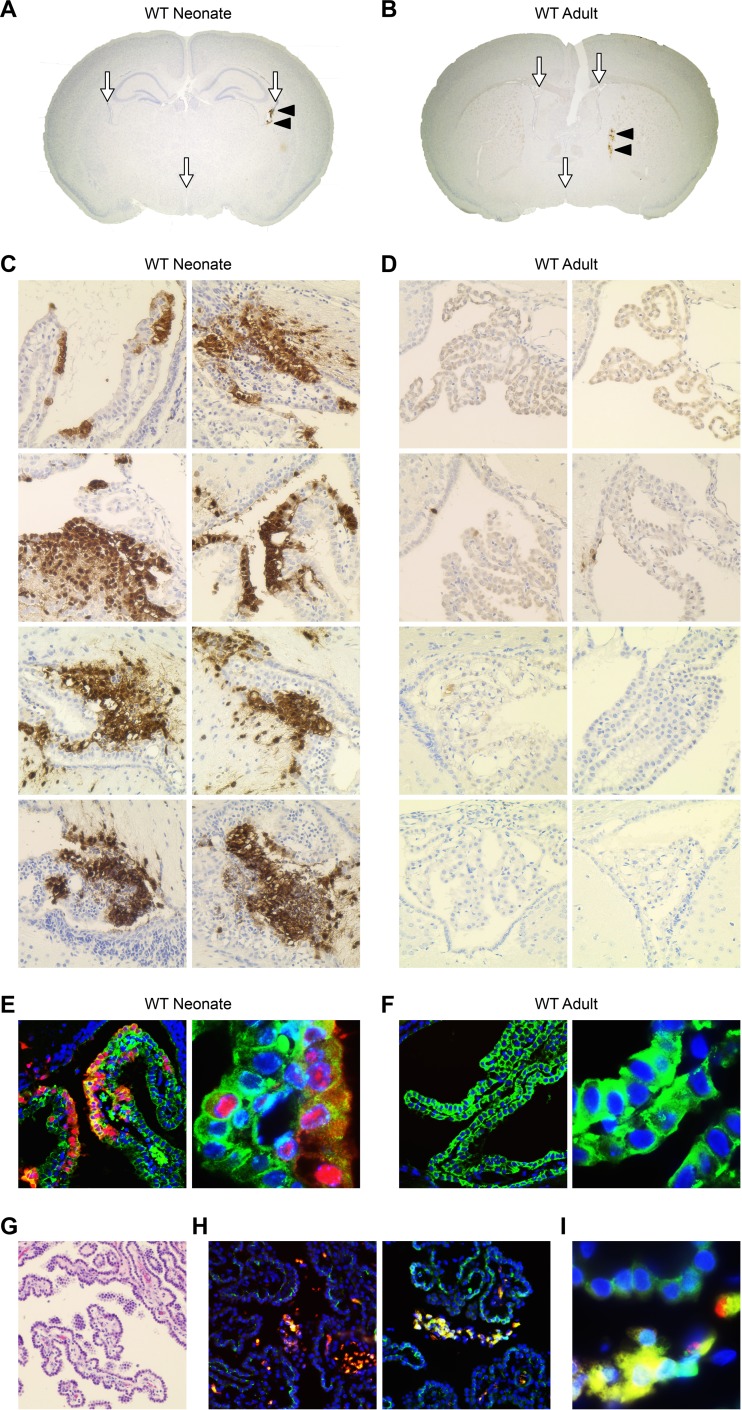
The choroid plexus (CP) is susceptible to HSV-1 early in infection of the newborn brain but not the adult brain. (A) Coronal section of WT newborn murine brain (original magnification, ×20) demonstrating the CSF-filled ventricles which contain the CP (white arrows) and HSV-1-positive cells in the CP, stained brown for HSV antigen (black arrowheads). (B) Coronal section of WT adult murine brain (original magnification, ×20) demonstrating ventricles with CP (white arrows) and HSV infection of brain parenchyma (black arrowheads). (C, D) Representative IHC for HSV antigen (original magnification, ×200) of neonatal WT mice (*n* = 4) inoculated i.c. with 10^3^ PFU of WT HSV-1 (C) and adult WT mice (*n* = 4) inoculated i.c. with 10^4^ PFU of WT HSV-1 (D) and perfused at 48 h postinfection. The CP in the newborn brain is positive (stained brown for HSV antigen) in all infected animals (rows) at different HSV-positive foci in the brain (columns). The adult CP is negative for HSV. (E, F) Representative IF of the newborn (E) and adult (F) murine CP for HSV (red), E-cadherin (green), and DAPI (blue) (original magnifications, ×200 [left] and ×1,000 [right]). The newborn mouse brain demonstrates colocalization of HSV and E-cadherin (merge, yellow). The adult murine CP is negative for HSV antigen. (G) Hematoxylin-and-eosin staining of HSV-infected human newborn CP. (H) Dual IF for HSV (red) and E-cadherin (green) in a human newborn case of HSV encephalitis (original magnification, ×200). (I) HSV-infected newborn human CP at higher magnification (×1,000). HSV-positive cells (red) are frequently detected in the CP fibrovascular stroma and in the E-cadherin-positive CP epithelium (merge, yellow).

To determine whether the choroid plexus was susceptible to HSV-1 early during infection of the newborn brain, neonatal mice were inoculated intracranially (i.c.) with 10^3^ PFU of wild-type (WT) HSV-1 and perfused 48 h postinfection for immunohistochemical (IHC) analysis. Direct intracranial inoculation circumvents the BBB to allow investigation of HSV neurotropism while controlling for possible differences in the BBB between the newborn and adult brain. HSV was robustly detected in the CP of all newborn mice (Fig. 1A and C) and only scantly detected in the brain parenchyma, including the site of inoculation (Fig. 1A). To determine whether the CP was similarly susceptible early in HSV infection of the adult brain, adult mice were inoculated with 10^4^ PFU of HSV-1 i.c. for IHC 48 h postinfection. In stark contrast to the newborn, the adult CP was not susceptible to HSV-1 infection (Fig. 1B and D), although viral antigen was detected in the adult brain parenchyma, consistent with prior reports (Fig. 1B) (14). This finding is additionally supported by prior work demonstrating a similar pattern of infection in the adult brain even at over 10-fold-higher doses of HSV-1 (15). Although the CP was not HSV positive in the adult, a few rare HSV-positive cells in neighboring ependymal cells and neurons were present (Fig. 1D), demonstrating that the virus did have access to the ventricles early in infection. Localization of HSV-1 to the CP in the newborn brain was confirmed using dual immunofluorescence (IF) for HSV antigen and E-cadherin, a specific marker for the specialized epithelial cells of the choroid plexus (Fig. 1E) (16). E-cadherin is not present in other neural cells or the distinct ependymal cells that line the ventricles of the brain (unpublished data). Consistent with IHC findings, HSV was not detected in E-cadherin-positive cells in the adult brain (Fig. 1F). Last, we demonstrated HSV targeting of the CP in a human newborn case of HSV encephalitis (Fig. 1G to I), with robust infection of E-cadherin-positive CP epithelial cells and, also, HSV-positive cells in the fibrovascular stroma of the CP (Fig. 1H and I). Taken together, these data demonstrate a difference between newborns and adults in the susceptibility of the choroid plexus to HSV-1 early in infection and confirmation of choroid plexus susceptibility to HSV in a human case of newborn encephalitis.

**FIG 2:**
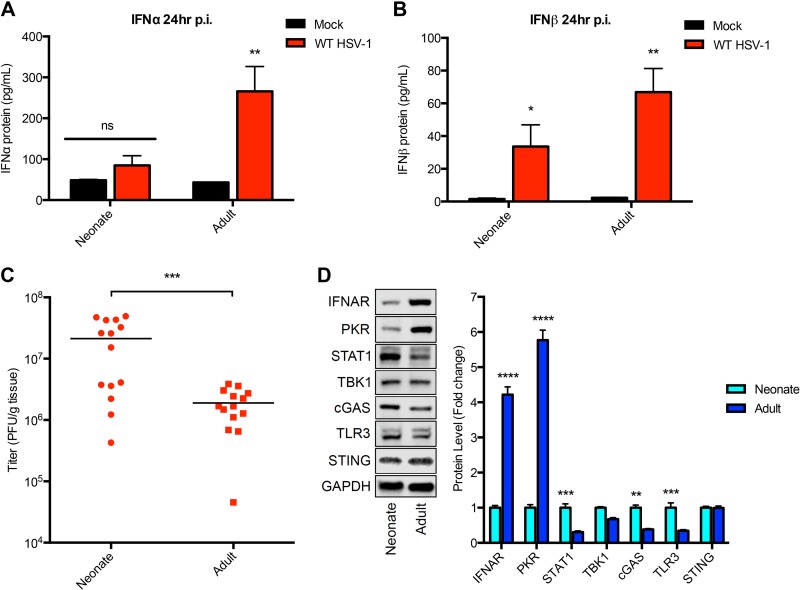
Reduced type I interferon production following HSV-1 infection, and IFN-α/β receptor (IFNAR) levels in the newborn murine brain compared to the levels in the adult brain. (A) Results of IFN-α ELISA of newborn and adult mouse brain homogenates either 24 h p.i. with 10^6^ PFU HSV-1 (*n* = 5, each age group) or 24 h after mock infection (*n* = 5, each age group). There was no statistically significant increase in IFN-α in the HSV-infected newborn brain compared to the level in the mock-infected control (*P* = 0.16). The HSV-infected adult brain had significantly increased IFN-α production compared to the level in the mock-infected adult brain at 24 h p.i. (*P* = 0.006). (B) IFN-β ELISA of brain homogenates either 24 h p.i. with 10^6^ PFU HSV-1 (*n* = 5, each age group) or 24 h after mock infection (*n* = 5, each age group). There was a statistically significant increase in IFN-β production following HSV-1 infection compared to its production in mock-infected controls in both the newborn (*P* = 0.04) and adult (*P* = 0.002) mouse brain. (C) HSV-1 titers in the brain at 24 h following i.c. inoculation with 10^6^ PFU virus. HSV-1 replication was significantly higher in the newborn brain than in the adult brain (mean titers of 2.12E7 PFU/g for the newborn and 1.90E6 PFU/g for the adult; *P* = 0.0008). (D) Representative immunoblots (left) and densitometry results (right) of brain homogenates from uninfected newborn and adult mice (*n* = 5 in each group). Protein levels of IFNAR and PKR were significantly lower in the newborn than in the adult brain. Basal levels of STAT1, cGAS, and TLR3 were higher in the newborn than in the adult brain. TBK1 and STING levels were similar in both age groups. *, *P* < 0.05; **, *P* < 0.01; ***, *P* < 0.001; ****, *P* < 0.001.

Prior studies have demonstrated that the HSV-1 entry receptor nectin-1 is expressed throughout the adult brain, including the choroid plexus (17). Nectin-1 is sufficient for HSV binding and entry (18), so this suggests a differential susceptibility of the choroid plexus to HSV-1 based on innate host factors. To determine components of the innate host response that may contribute to susceptibility of the newborn CP, we characterized the early type I IFN response in the newborn brain in comparison to that in the adult brain. Following i.c. inoculation with HSV-1, enzyme-linked immunosorbent assay (ELISA) of brain homogenates demonstrated no increased production of alpha interferon (IFN-α) at 24 h postinfection in the newborn brain compared to the level in mock-infected controls (Fig. 2A). In contrast, IFN-α production 24 h postinfection was readily detected in the adult brain, consistent with prior reports (19). Type I IFN production was not completely absent in the newborn brain, since IFN-β was detected in both age groups, although at a lower level in the newborn than in the adult (Fig. 2B). Interestingly, the levels of production of type I IFNs were lower in the newborn brain despite higher viral loads than in the adult brain at the same time point (Fig. 2C).

To determine whether the reduced interferon production in the newborn brain was a result of global down-regulation of the type I IFN response or specific components of the pathway, we investigated across the two age groups the basal levels of several host proteins previously shown to play a critical role in the type I IFN response to HSV infection (20–24). Surprisingly, IFN-α/β receptor (IFNAR) levels were dramatically lower (more than fourfold) in the newborn brain than in the adult brain (Fig. 2D). Similarly, the levels of the double-stranded RNA (dsRNA)-dependent protein kinase R (PKR) were also significantly reduced in the newborn brain. The reduced levels of IFNAR and PKR in the newborn brain were not a result of global down-regulation of the type I IFN pathway, since STAT1 and cGAS were present at significantly higher levels in the newborn brain than in the adult brain. The increased level of Toll-like receptor 3 (TLR3) in the newborn brain is consistent with a physiologic role of TLR3 in neurodevelopment and has previously been described by others (25).

**FIG 3:**
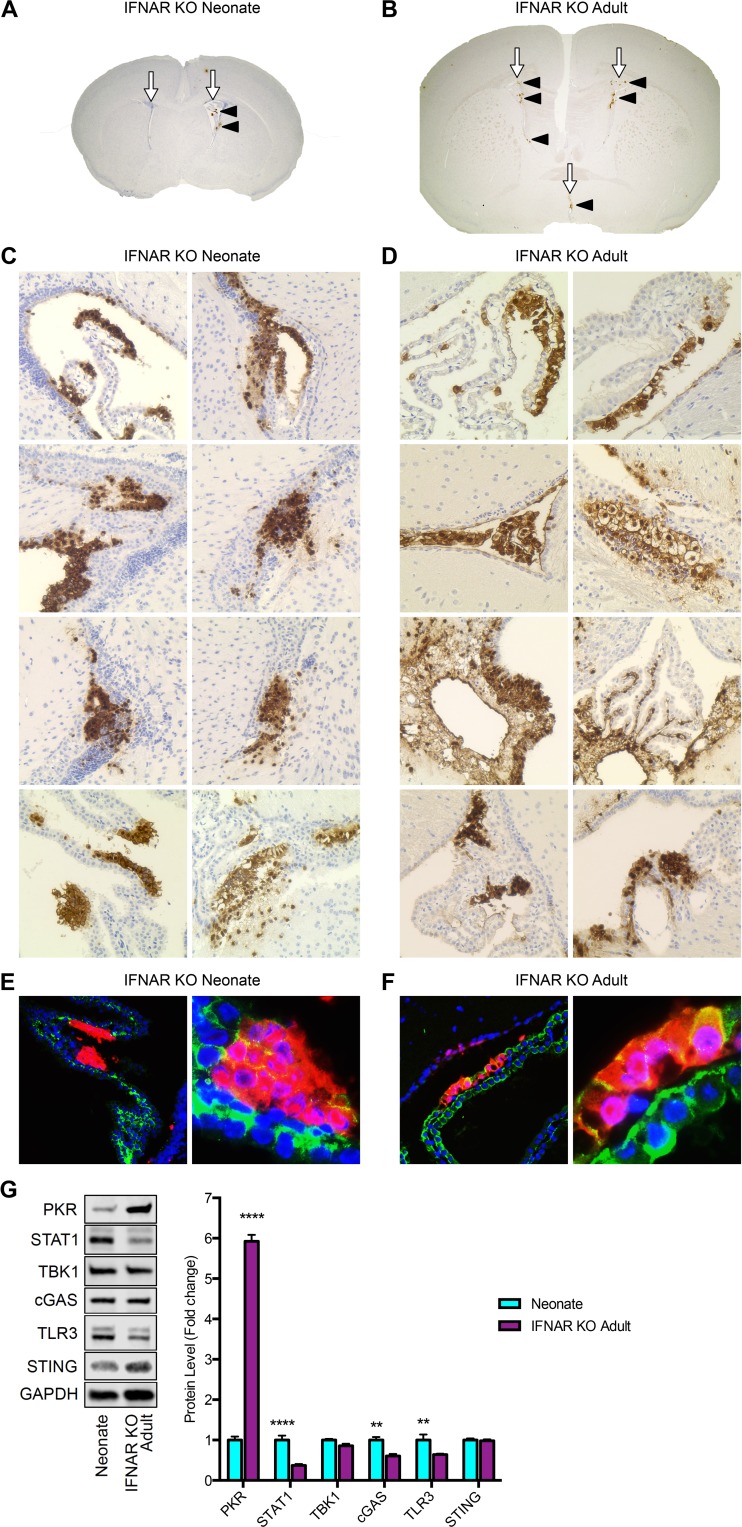
Genetic ablation of IFNAR restores the early susceptibility of the choroid plexus to HSV infection in the adult. (A) Coronal section of IFNAR KO newborn murine brain (original magnification, ×20) demonstrating the ventricles with CP (white arrows) and HSV-1-positive cells in the CP, stained brown for HSV antigen (black arrowheads). (B) Coronal section of IFNAR KO adult murine brain (original magnification, ×20) demonstrating ventricles with CP (white arrows) and HSV infection of CP (black arrowheads). (C, D) Representative IHC for HSV antigen (original magnification, ×200) of newborn IFNAR KO mice (*n* = 4) inoculated i.c. with 10^3^ PFU of WT HSV-1 (C) and adult IFNAR KO mice (*n* = 4) inoculated i.c. with 10^4^ PFU of WT HSV-1 (D) and perfused at 48 h p.i. The CP in IFNAR KO newborns was positive in all infected animals (rows) at different HSV foci in the brain (columns). The adult IFNAR KO CP was robustly positive for HSV in all infected animals. (E, F) Representative IF of IFNAR KO newborn (E) and IFNAR KO adult (F) murine choroid plexus for HSV (red), E-cadherin (green), and DAPI (blue) (original magnifications, ×200 [left] and ×1,000 [right]). IFNAR KO newborn CP and IFNAR KO adult CP both demonstrate colocalization of HSV with E-cadherin (merge, yellow). (G) Representative immunoblots (left) and densitometry results (right) of brain homogenates from uninfected WT newborn and IFNAR KO adult mice (*n* = 5 in each group). Protein levels of PKR remained significantly lower in the WT newborn brain than in the IFNAR KO adult brain. Basal levels of STAT1, cGAS, and TLR3 remained higher in the newborn brain than in the IFNAR KO adult brain. TBK1 and STING levels were similar in both age groups. *, *P* < 0.05; **, *P* < 0.01; ***, *P* < 0.001; ****, *P* < 0.001.

IFNAR is ligated by IFN-α/β to stimulate the host interferon response and, ultimately, produce several antiviral interferon-stimulated genes (ISGs) (26). In the adult, signaling through IFNAR reduces viral titers and improves survival in mice (5). To determine the contribution of IFNAR levels to CP susceptibility in HSV-1 infection, newborn and adult IFNAR knockout (KO) mice were inoculated with HSV-1 as described in Materials and Methods. Consistent with our studies in WT newborn mice (Fig. 1), the CP was susceptible early in infection of IFNAR KO newborn mice (Fig. 3A and C). Genetic ablation of IFNAR in the adult restored susceptibility of the choroid plexus to HSV-1 (Fig. 3B and D). Additionally, infection of the CP early in infection of IFNAR KO adults demonstrated similar viral access to the ventricles in both age groups following i.c. inoculation, but establishment of infection was dependent on host factors. Across both age groups of mice deficient in IFNAR, HSV consistently colocalized with E-cadherin (Fig. 3E and F). These data demonstrate that IFNAR restricts HSV-1 neurotropism from the choroid plexus and that reduced levels of IFNAR contribute to the early susceptibility of the choroid plexus in the newborn brain.

Similar to several mediators of the type I interferon response, IFNAR participates in feedback loops to regulate its own expression and the expression of many other ISGs (27). To determine whether the altered levels of PKR, STAT1, cGAS, and TLR3 in the naive newborn brain compared to their levels in the adult brain (Fig. 2D) were the result of significantly reduced IFNAR levels in the newborn or a result of developmental age, we compared the basal protein levels in the WT newborn brain to the basal levels in the IFNAR KO adult brain (Fig. 3G). Interestingly, deletion of IFNAR in the adult did not restore the levels of PKR, STAT1, cGAS, or TLR3 to newborn brain levels. This suggests that the decreased IFNAR level in the WT newborn brain does not account for the significantly decreased level of PKR and suggests an IFN-independent down-regulation of PKR in the newborn brain. This may have important implications for disease severity in the newborn, since PKR has been well described to contribute to restricting viral replication and improving survival in adult models of encephalitis (20).

In summary, we report that the choroid plexus is susceptible to HSV-1 early in infection of the newborn murine brain but is protected from infection in the adult. We also provide the first description of HSV infection of the CP in a newborn human case of HSV encephalitis. Compared to the adult mouse brain, the newborn mouse brain demonstrated reduced IFN production following HSV-1 infection. IFNAR is present at a significantly higher level in the adult mouse brain than in the newborn brain, and ablation of IFNAR in the adult mouse brain restored susceptibility of the CP to HSV-1 infection, similar to the WT newborn. Finally, genetic ablation of IFNAR in the adult mouse did not decrease PKR levels, demonstrating a likely IFN-independent down-regulation of PKR in the newborn mouse brain.

## MATERIALS AND METHODS

### Viruses and cells.

The WT HSV-1 strain 17+ virus was kindly provided by Richard Thompson, University of Cincinnati, Cincinnati, OH and was previously described (28). Vero cells were cultured in DMEM plus 10% (vol/vol) FBS and 1% penicillin-streptomycin and were used for propagation and titration of virus. Plaque titrations were performed by standard methods. Comparisons of viral titers between the two different age groups were done by Student’s *t* test.

### Murine HSV encephalitis model.

Animal care and use in this study were in accordance with institutional and NIH guidelines, and all studies were approved by the Northwestern University Animal Care and Use Committee. The mouse strains used have been previously described, including the 129S2 (WT) and IFN-α/β receptor knockout (IFNAR KO) mice on the 129S2 genetic background (26). Pups were inoculated at 7 days of age, which from an immunologic perspective corresponds most closely to humans at birth (3). Virus was diluted to the appropriate dose for each experiment with phosphate-buffered saline (PBS) containing 1% inactivated calf serum and 0.1% glucose (PBS-GCS). PBS-GCS without virus was used for mock-infected controls.

### Immunohistochemistry and dual immunofluorescence.

Seven-day-old newborn mice were inoculated i.c. with 10^3^ PFU of HSV-1 strain 17+, and 10-week-old adult mice were inoculated i.c. with 10^4^ PFU HSV-1 strain 17+. A positive displacement syringe with a 26-gauge needle and a needle guard was used to inoculate a 5-µl total volume into the brain. The needle was placed in the approximate region of the hippocampus, equidistant between the lambda and bregma, through the left parietal bone lateral to the sagittal suture. The placement of the needle was confirmed in paraffin sections. Anesthetized mice were subjected to intracardiac perfusion with 4% formaldehyde and subsequently embedded in paraffin. Four-micrometer-thick sections were mounted on glass slides. Antigen retrieval was performed manually using citric acid-based solution (Vector Laboratories) at 95°C for 10 min. IHC staining was performed with anti-HSV antigen (Dako) diluted 1:5,000. Horseradish peroxidase (HRP)-labeled secondary antibodies were visualized after treatment with diaminobenzidine (DAB; Vector Laboratories). Slides were counterstained with Gill’s hematoxylin and imaged with the EVOS XL core cell imaging system. For dual immunofluorescence imaging, slides were stained with anti-HSV antigen antibody (1:10,000; Dako) and anti-E-cadherin antibody (1:200; BD) and the nuclear stain DAPI (Life Technologies). Secondary antibodies conjugated with Alexa Fluor 568 (Life Technologies) and Alex Flour 488 (Jackson ImmunoResearch) were used for visualization.

### Immunoblots.

Brain tissue samples were whole-organ homogenates from neonatal and adult mice. Western blots were performed on cell lysates using a 1:1,000 dilution of antibodies against IFNAR (Abcam), PKR (Abcam), STAT1 (Cell Signaling), TBK1 (Cell Signaling), cGAS (Millipore), TLR3 (Cell Signaling), and STING (Cell Signaling) and a 1:10,000 dilution of anti-glyceraldehyde-3-phosphate dehydrogenase (GAPDH) antibody (Abcam) as a loading control. Immunoblots were visualized using the LI-COR Odyssey system. Statistical analysis of densitometry was performed using a two-way analysis of variance (ANOVA) and the Holm-Sidak multiple comparison test.

### ELISA.

Neonatal and adult mice were inoculated i.c. with 10^6^ PFU of HSV-1 strain 17+ in a 20-µl total volume and sacrificed at 24 h postinoculation. ELISA of IFN-α and IFN-β (PBL Assay Science) was performed on brain homogenates, and the results were read at 450 nm after treatment with tetramethyl-benzidine (TMB).

### Human samples.

Permission for the use of human postmortem tissue for this study was obtained from the Ann & Robert H. Lurie Children’s Hospital of Chicago Privacy Board, in accordance with the U.S. Code of Federal Regulations 45 CFR 46.160 and 164 (29, 30). Samples of brain tissue were obtained at autopsy from a 7-week-old male who presented at 1 month of age with symptoms of encephalitis, confirmed by HSV-positive CSF and brain tissue.

### Statistics.

All results are expressed as the mean ± standard error of the mean (SEM). Statistical analyses were performed using Prism 5.01 (GraphPad Software). For ELISA and titer assay, the two-tailed, unpaired Student *t* test was used was used for two-group comparisons. For multiple comparisons of densitometry data, a two-way ANOVA with Holm-Sidak’s multiple comparison test was used. In all instances, probability values of less than 0.05 were considered significant and are described in the figure legends.

### Study approval.

Animal care and use in this study were in accordance with institutional and NIH guidelines, and all studies were approved by the Northwestern University Animal Care and Use Committee. Permission for the use of human postmortem tissue for this study was obtained from the Ann & Robert H. Lurie Children’s Hospital of Chicago Privacy Board, in accordance with U.S. Code of Federal Regulations 45 CFR 46.160 and 164 (29, 30).

## References

[1] Langenberg AG, Corey L, Ashley RL, Leong WP, Straus SE 1999 A prospective study of new infections with herpes simplex virus type 1 and type 2. Chiron HSV Vaccine Study Group. N Engl J Med 341:1432–1438. doi:10.1056/NEJM199911043411904. 10547406

[2] Kimberlin DW, Lin CY, Jacobs RF, Powell DA, Frenkel LM, Gruber WC, Rathore M, Bradley JS, Diaz PS, Kumar M, Arvin AM, Gutierrez K, Shelton M, Weiner LB, Sleasman JW, de Sierra TM, Soong SJ, Kiell J, Lakeman FD, Whitley RJ 2001 Natural history of neonatal herpes simplex virus infections in the acyclovir era. Pediatrics 108:223–229. doi:10.1542/peds.108.2.223. 11483781

[3] Adkins B, Leclerc C, Marshall-Clarke S 2004 Neonatal adaptive immunity comes of age. Nat Rev Immunol 4:553–564. doi:10.1038/nri1394. 15229474

[4] Wilcox DR, Wadhwani NR, Longnecker R, Muller WJ 2015 Differential reliance on autophagy for protection from HSV encephalitis between newborns and adults. PLoS Pathog 11:e1004580. doi:10.1371/journal.ppat.1004580. 25569138 PMC4287605

[5] Wang JP, Bowen GN, Zhou S, Cerny A, Zacharia A, Knipe DM, Finberg RW, Kurt-Jones EA 2012 Role of specific innate immune responses in herpes simplex virus infection of the central nervous system. J Virol 86:2273–2281. doi:10.1128/JVI.06010-11. 22171256 PMC3302371

[6] Wilcox DR, Muller WJ, Longnecker R 2015 HSV targeting of the host phosphatase PP1α is required for disseminated disease in the neonate and contributes to pathogenesis in the brain. Proc Natl Acad Sci U S A 112:E6937–E6944 doi:10.1073/pnas.1513045112. 26621722 PMC4687582

[7] Kopp SJ, Banisadr G, Glajch K, Maurer UE, Grünewald K, Miller RJ, Osten P, Spear PG 2009 Infection of neurons and encephalitis after intracranial inoculation of herpes simplex virus requires the entry receptor nectin-1. Proc Natl Acad Sci USA 106:17916–17920. doi:10.1073/pnas.0908892106. 19805039 PMC2764878

[8] Feuer R, Mena I, Pagarigan RR, Harkins S, Hassett DE, Whitton JL 2003 Coxsackievirus B3 and the neonatal CNS: the roles of stem cells, developing neurons, and apoptosis in infection, viral dissemination, and disease. Am J Pathol 163:1379–1393. doi:10.1016/S0002-9440(10)63496-7. 14507646 PMC1868316

[9] Dando SJ, Mackay-Sim A, Norton R, Currie BJ, St John JA, Ekberg JA, Batzloff M, Ulett GC, Beacham IR 2014 Pathogens penetrating the central nervous system: infection pathways and the cellular and molecular mechanisms of invasion. Clin Microbiol Rev 27:691–726. doi:10.1128/CMR.00118-13. 25278572 PMC4187632

[10] Meeker RB, Williams K, Killebrew DA, Hudson LC 2012 Cell trafficking through the choroid plexus. Cell Adh Migr 6:390–396. doi:10.4161/cam.21054. 22902764 PMC3496674

[11] Lehtinen MK, Zappaterra MW, Chen X, Yang YJ, Hill AD, Lun M, Maynard T, Gonzalez D, Kim S, Ye P, D’Ercole AJ, Wong ET, LaMantia AS, Walsh CA 2011 The cerebrospinal fluid provides a proliferative niche for neural progenitor cells. Neuron 69:893–905. doi:10.1016/j.neuron.2011.01.023. 21382550 PMC3085909

[12] Marques F, Sousa JC, Coppola G, Falcao AM, Rodrigues AJ, Geschwind DH, Sousa N, Correia-Neves M, Palha JA 2009 Kinetic profile of the transcriptome changes induced in the choroid plexus by peripheral inflammation. J Cereb Blood Flow Metab 29:921–932. doi:10.1038/jcbfm.2009.15. 19240744

[13] Stolp HB, Turnquist C, Dziegielewska KM, Saunders NR, Anthony DC, Molnár Z 2011 Reduced ventricular proliferation in the foetal cortex following maternal inflammation in the mouse. Brain 134:3236–3248. doi:10.1093/brain/awr237. 21964917 PMC3212715

[14] Kopp SJ, Banisadr G, Glajch K, Maurer UE, Grünewald K, Miller RJ, Osten P, Spear PG 2009 Infection of neurons and encephalitis after intracranial inoculation of herpes simplex virus requires the entry receptor nectin-1. Proc Natl Acad Sci U S A 106:17916–17920. doi:10.1073/pnas.0908892106. 19805039 PMC2764878

[15] Braun E, Zimmerman T, Hur TB, Reinhartz E, Fellig Y, Panet A, Steiner I 2006 Neurotropism of herpes simplex virus type 1 in brain organ cultures. J Gen Virol 87:2827–2837. doi:10.1099/vir.0.81850-0. 16963740

[16] Menheniott TR, Charalambous M, Ward A 2010 Derivation of primary choroid plexus epithelial cells from the mouse. Methods Mol Biol 633:207–220. doi:10.1007/978-1-59745-019-5_15. 20204630

[17] Shukla D, Scanlan PM, Tiwari V, Sheth V, Clement C, Guzman-Hartman G, Dermody TS, Valyi-Nagy T 2006 Expression of nectin-1 in normal and herpes simplex virus type 1-infected murine brain. Appl Immunohistochem Mol Morphol 14:341–347. doi:10.1097/00129039-200609000-00014. 16932027

[18] Geraghty RJ, Krummenacher C, Cohen GH, Eisenberg RJ, Spear PG 1998 Entry of alphaherpesviruses mediated by poliovirus receptor-related protein 1 and poliovirus receptor. Science 280:1618–1620. doi:10.1126/science.280.5369.1618. 9616127

[19] Menachery VD, Pasieka TJ, Leib DA 2010 Interferon regulatory factor 3-dependent pathways are critical for control of herpes simplex virus type 1 central nervous system infection. J Virol 84:9685–9694. doi:10.1128/JVI.00706-10. 20660188 PMC2937762

[20] Leib DA, Machalek MA, Williams BR, Silverman RH, Virgin HW 2000 Specific phenotypic restoration of an attenuated virus by knockout of a host resistance gene. Proc Natl Acad Sci U S A 97:6097–6101. doi:10.1073/pnas.100415697. 10801979 PMC18564

[21] Pasieka TJ, Cilloniz C, Carter VS, Rosato P, Katze MG, Leib DA 2011 Functional genomics reveals an essential and specific role for Stat1 in protection of the central nervous system following herpes simplex virus corneal infection. J Virol 85:12972–12981. doi:10.1128/JVI.06032-11. 21994441 PMC3233176

[22] Mørk N, Kofod-Olsen E, Sørensen KB, Bach E, Ørntoft TF, Østergaard L, Paludan SR, Christiansen M, Mogensen TH 2015 Mutations in the TLR3 signaling pathway and beyond in adult patients with herpes simplex encephalitis. Genes Immun 16:552–566. doi:10.1038/gene.2015.46. 26513235

[23] Li XD, Wu J, Gao D, Wang H, Sun L, Chen ZJ 2013 Pivotal roles of cGAS-cGAMP signaling in antiviral defense and immune adjuvant effects. Science 341:1390–1394. doi:10.1126/science.1244040. 23989956 PMC3863637

[24] Parker ZM, Murphy AA, Leib DA 2015 Role of the DNA sensor STING in protection from lethal infection following corneal and intracerebral challenge with herpes simplex virus 1. J Virol 89:11080–11091. doi:10.1128/JVI.00954-15. 26311879 PMC4621135

[25] Kaul D, Habbel P, Derkow K, Krüger C, Franzoni E, Wulczyn FG, Bereswill S, Nitsch R, Schott E, Veh R, Naumann T, Lehnardt S 2012 Expression of Toll-like receptors in the developing brain. PLoS One 7:e37767. doi:10.1371/journal.pone.0037767. 22666391 PMC3364272

[26] Müller U, Steinhoff U, Reis LF, Hemmi S, Pavlovic J, Zinkernagel RM, Aguet M 1994 Functional role of type I and type II interferons in antiviral defense. Science 264:1918–1921. doi:10.1126/science.8009221. 8009221

[27] Fuchs SY 2013 Hope and fear for interferon: the receptor-centric outlook on the future of interferon therapy. J Interferon Cytokine Res 33:211–225. doi:10.1089/jir.2012.0117. 23570388 PMC3624693

[28] Bolovan CA, Sawtell NM, Thompson RL 1994 ICP34.5 mutants of herpes simplex virus type 1 strain 17syn+ are attenuated for neurovirulence in mice and for replication in confluent primary mouse embryo cell cultures. J Virol 68:48–55. 8254758 10.1128/jvi.68.1.48-55.1994 PMC236262

[29] Code of Federal Regulations 45 CFR 160. Title 45. Public welfare. Subtitle A. Department of Health and Human Services. http://www.ecfr.gov/cgi-bin/text-idx?tpl=/ecfrbrowse/Title45/45cfr160_main_02.tpl.

[30] Code of Federal Regulations 45 CFR 164. Title 45. Public welfare. Subtitle A. Department of Health and Human Services. http://www.ecfr.gov/cgi-bin/text-idx?tpl=/ecfrbrowse/Title45/45cfr164_main_02.tpl.

